# Functions of RNA-Binding Proteins in Cardiovascular Disease

**DOI:** 10.3390/cells12242794

**Published:** 2023-12-08

**Authors:** Grégoire Ruffenach, Lejla Medzikovic, Wasila Sun, Jason Hong, Mansoureh Eghbali

**Affiliations:** 1 Department of Anesthesiology, Division of Molecular Medicine, David Geffen School of Medicine, University of California, Los Angeles, CA 90095, USAsunwasila@gmail.com (W.S.); 2 Department of Pulmonary and Critical Care Medicine, David Geffen School of Medicine, University of California, Los Angeles, CA 90095, USA

**Keywords:** RNA binding protein, cardiovascular diseases, pathogenesis

## Abstract

Gene expression is under tight regulation from the chromatin structure that regulates gene accessibility by the transcription machinery to protein degradation. At the transcript level, this regulation falls on RNA-binding proteins (RBPs). RBPs are a large and diverse class of proteins involved in all aspects of a transcript’s lifecycle: splicing and maturation, localization, stability, and translation. In the past few years, our understanding of the role of RBPs in cardiovascular diseases has expanded. Here, we discuss the general structure and function of RBPs and the latest discoveries of their role in pulmonary and systemic cardiovascular diseases.

## 1. Introduction

Cardiovascular diseases affect both circulations—systemic and pulmonary—and all cardiac chambers; and they are the main cause of death worldwide [1].

The most common pulmonary and systemic vascular diseases are pulmonary hypertension (PH) and atherosclerosis, respectively. Pulmonary hypertension is characterized by the increased blood pressure within the pulmonary circulation. Among the different forms of PH, the mostly commonly studied is pulmonary arterial hypertension (PAH). PAH arises from the loss of pulmonary vascular bed and narrowing of the lumen of pulmonary arteries. Together, they lead to right ventricular maladaptive hypertrophy, and over time to right heart failure and death [2]. Atherosclerosis, the most common systemic vascular disease, is the hardening and narrowing of arteries due to neointima formation, immune cell infiltration, and lipid accumulation. It is a cornerstone of cardiovascular mortality and morbidity [3].

Cardiac disease develops independently or secondarily to vascular disease. In both cases they are the cause of death. Among them, heart failure (HF), myocardial infarction (MI), cardiac fibrosis, and cardiomyopathies are the most common. HF is characterized by changes in the size, shape, and function of the heart that ultimately lead to decreased cardiac output. MI due to atherosclerotic or thrombotic occlusion of coronary arteries leads to ischemia in the myocardium and cardiomyocyte death. Rapid reperfusion is the cornerstone of current MI treatment, but restoring blood flow leads to further damage to the myocardium by causing ischemia-reperfusion injury (IRI) [4]. Cardiac fibrosis is excessive extracellular matrix production and contributes to numerous cardiac disorders by impairing myocardial compliance, contractility, and electrical conductance [5]. Cardiomyopathies are myocardial disorders characterized by structural and functional abnormalities [6]. In dilated cardiomyopathy (DCM), the left ventricle is enlarged and exhibits poor contractility, causing DCM to be a major HF risk factor.

Independent of their underlying causes—genetic or environmental—cardiovascular diseases all propel profound gene expression modifications. Gene expression is under tight regulation from the chromatin structure that regulates gene accessibility to protein degradation. At the transcript level, this regulation falls on RNA-binding proteins (RBPs). RBPs are a large and diverse class of proteins involved in all aspects of a transcript’s lifecycle: splicing and maturation, localization, stability, and translation. Because RBPs regulate a large part of a cell’s transcriptome, they emerged as a key player in cardiovascular diseases. RBPs have also attracted attention for their therapeutic potential, and small molecules are developed to modify their function and counteract their negative role in disease development and progression.

In this review, we discuss the general structure and function of RBPs, the latest discoveries of their role in pulmonary and systemic cardiovascular diseases (Table 1), and their therapeutic potential.

## 2. RNA-Binding Protein Structure and Function

### 2.1. RNA-Binding Protein Function

**Figure 1:**
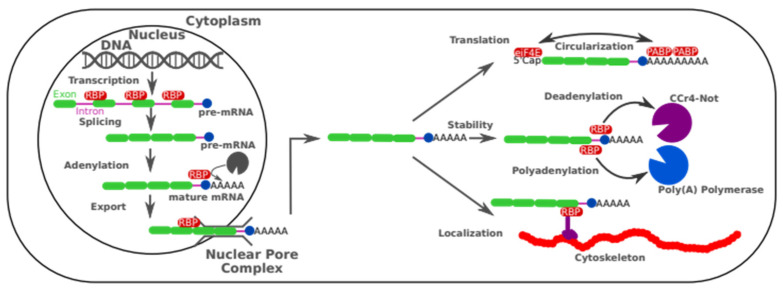
Main function of RNA-binding Protein. In the nucleus, once transcribed, the pre-mRNA is maturated by the binding of RBP at a specific site to recruit the spliceosome and remove the introns. Then, RBPs will recognize the poly(A) site and recruit the poly(A) polymerase to add the poly(A) tail. Finally, the mature mRNA will be exported from the nucleus into the cytoplasm with the help of the RBP complex that will bind both the mature mRNA and the Nuclear pore. In the cytoplasm, the poly(A) tail will be covered with poly(A) binding protein to protect it from exonuclease. The PABP will also interact with eiF4E bound to the 5′Cap and circularize it to improve the translation efficiency. Specific RBP complexes will also recruit dyneins to move the mRNA to a specific location. Finally, RBPs will recruit the Ccr4-Not deadenylase complex to start the degradation process.

RNA-binding proteins have evolved to regulate all aspects of the mRNA life cycle: splicing, expression, localization, and stability (Figure 1).

#### 2.1.1. Splicing

Once transcribed, pre-mRNAs are spliced into mature mRNA. At first glance, intron splicing is a very inefficient strategy: transcribing sequences only to be removed at the next stage by the large and intricate machinery that is the spliceosome. But, from an evolutionary standpoint, introns have been an essential part of genetic coding since the RNA world [7]. Today, the spliceosome is a highly regulated plastic RNA-protein enzymatic complex. The spliceosome catalyzes intronic exclusion at constitutive splicing sites as well as at alternative splicing sites. As opposed to constitutive splicing, which is a straightforward mechanism, alternative splicing is a highly context-dependent mechanism regulated by the RBP recognition of motifs [8]. One motif can be recognized differently by any given RBP depending on the protein complex it is in. This motif can also be recognized by different RBPs. Finally, the localization of the motif on the mRNA influences the function of the RBP bound to it: when the binding is within an exon, the adjacent intron is included; when the binding is within the intron, it is spliced. However, these are general rules, they can be broken. We now know at least twenty-six RBP splicing regulators but their exact mechanism of action remains elusive [8].

#### 2.1.2. Expression

Polyadenylation, the addition of a poly(A) tail in 3′ is part of the nuclear maturation process of mRNA before export and cytoplasmic translation. The tail is covered and protected from degradation with poly(A) binding protein (PABP). PABP also interacts with eIF4E bound to the 5′-Cap to circularize the mRNA, a critical step to increase translation efficiency. The circularization promotes the recycling of post-termination ribosomes for translation reinitiation. Cytoplasmic regulation of the length of the poly(A) tail is thus a highly efficient mechanism to regulate mRNA translation [9].

During the first developmental stages, gene expression relies only on maternal RNA, as transcription is absent. During that time, RBPs orchestrate gene expression. In immature oocytes, the RBP CPEB interacts with the U-rich 3′UTR element and represses translation. In mature oocytes, after fertilization, CPEB promotes cytoplasmic adenylation and translation [10]. Cytoplasmic adenylation is not specific to oocytes. It is a widespread method of gene expression regulation used by yeast, mice, and humans after nuclear transcription starts [11].

#### 2.1.3. Sub-Cellular Localization

Tightly linked to translation in eukaryotic cells, targeted mRNA localization is integral to gene expression regulation [12]. Asymmetric mRNA distribution was first observed in oocytes, where it is essential to establish cell polarity. Since then, it has been observed in many eukaryotic cells, like neurons and migratory fibroblasts. Localization of a mRNA is cost-efficient compared to localizing the hundreds of proteins generated by a single mRNA. Hence, mRNA localization became a fundamental process to fine-tune gene expression and regulate cell phenotypes.

Different mechanisms exist to localize mRNAs: active transport, protection from degradation at a specific locus, and passive diffusion. Passive diffusion is only efficient in small cells. Hence, only bacteria rely on this process [13]. Active transport and mRNA protection are both carried out by RBPs. Active transport is carried by RBPs through the recognition of a specific motif. Once bound to mRNAs, RBPs recruit motor proteins and move the mRNAs along the myosin chain [14]. The mRNA protection mechanism is based on the specific localization of an RBP. At the site where the RBP is, the mRNA will be bound by it, protected from degradation, and translated. In any other locus, the absence of the protective RBP will lead to rapid mRNA degradation [12].

#### 2.1.4. Stability

The stability of mRNA is regulated by the length of the poly(A) tail. Experimental evidence supports that deadenylation is the first limiting step of mRNA degradation, and PABP that covers the poly(A) tail protects it from exonuclease in the vicinity. RBPs such as Tristetraprolin (TTP) or Pumilio bind to specific AU-rich elements within the 3′UTR and recruit the deadenylation complex, Ccr4-Not that leads to translation silencing or degradation [15].

Increasing poly(A) tail length stabilizes the mRNA and favors translation. Cytoplasmic polyadenylation of mRNA is known to occur, but the constant transcription of new mRNA and the localization-specific adenylation and deadenylation process make it difficult to investigate in detail [16]. Nonetheless, it is clear that poly(A) tail length is a cornerstone of gene expression regulation in eukaryotic cells.

The aforementioned examples show the central role of RBPs in gene expression regulation and underline the role RBP dysregulation may play in cardiovascular disease development. The ever-growing number of RNA-binding proteins involved in cardiovascular disease cannot be exhaustively covered. In the following sections, we present the implication of RBPs in a wide range of cardiovascular diseases, both systemic and pulmonary, through a few selected examples.

### 2.2. RNA-Binding Protein Structure

RNA-binding proteins (RBP) use a modular structure, a fit-for-purpose combination of RNA-binding Domains (RBDs). RBDs are well-defined structures that recognize a ribonucleotide sequence, a structural motif, or both with weak affinity [17]. The assembly of multiple RBDs within one RBP creates versatile binding surfaces with high affinity and specificity that accommodate the dynamic equilibrium between binding and unbinding. Furthermore, each RBD can evolve independently and adapt to poorly conserved motifs [18,19].

Several canonical RBDs exist, each with a specific method to recognize their targets. A detailed classification of RBDs and their mean-to-bind motifs are available in numerous curated reviews [20,21,22]. Here are a few examples of their great variety.

**Figure 2:**
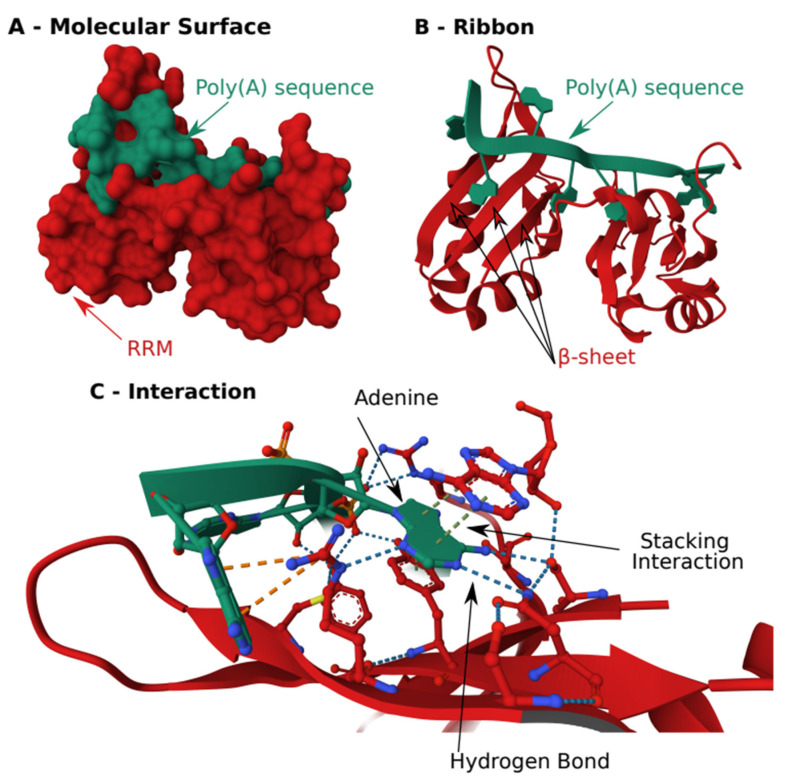
**Structure of the RNA Recognition Motif of the Human Poly.** (**A**) Binding Protein in complex with a poly(A). (**A**) Schematic of the molecular surface. (**B**) Schematic of arrangement of β-sheets and α-helix of the RRM to recognize the poly(A). (**C**) Close view of the stacking interaction where the adenine is stacked between two aromatic side chains of amino acid. The specificity of the interaction is obtained by the hydrogen bond on the side of the stack. The structure was obtained from the protein data bank, structure: 1CVJ.

The most common RBD is the RNA recognition motif (RRM). More than 9000 RRMs have been described [23]. RRMs use aromatic acids of β-sheets to create a stacking interaction with nucleobases. This stacking interaction between aromatic amino acid and nucleobase creates the single-strand RNA recognition of the RRM but does not provide sequence-specificity. To achieve specificity, the RRM motif relies on other side chains and residues in the immediate C-terminal of the RRM [24] (Figure 2).

**Figure 3:**
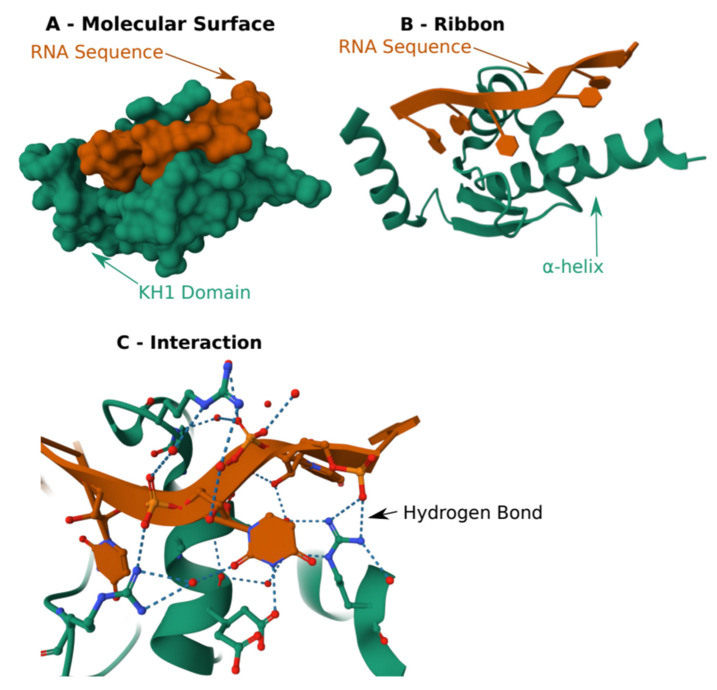
**Structure of KH1 domain of MEX-3C E3 ubiquitin Ligase in complex with RNA**. (**A**) Schematic of the molecular surface. (**B**) Schematic of arrangement of B-sheets and a-helix to recognize RNA. (**C**) Close view of the hydrogen bonds that create the binding. The structure was obtained from the protein data bank, structure: 5WWW.

Another example is the KH domain. Unlike the RRM domain, the KH domain is built around three α-helices and two β-sheets. As opposed to many other RBDs, KH does not use stacking interactions to recognize RNA. It uses the protein backbone of the α-helix to recognize its targets through hydrogen bonds, electrostatic interaction, and shape complementarity3. For example, in some cases, the KH domain recognizes an adenine backbone by hydrogen bonded between the protein backbone and the adenines, recreating the Watson–Crick base pairing pattern [25] (Figure 3).

**Figure 4:**
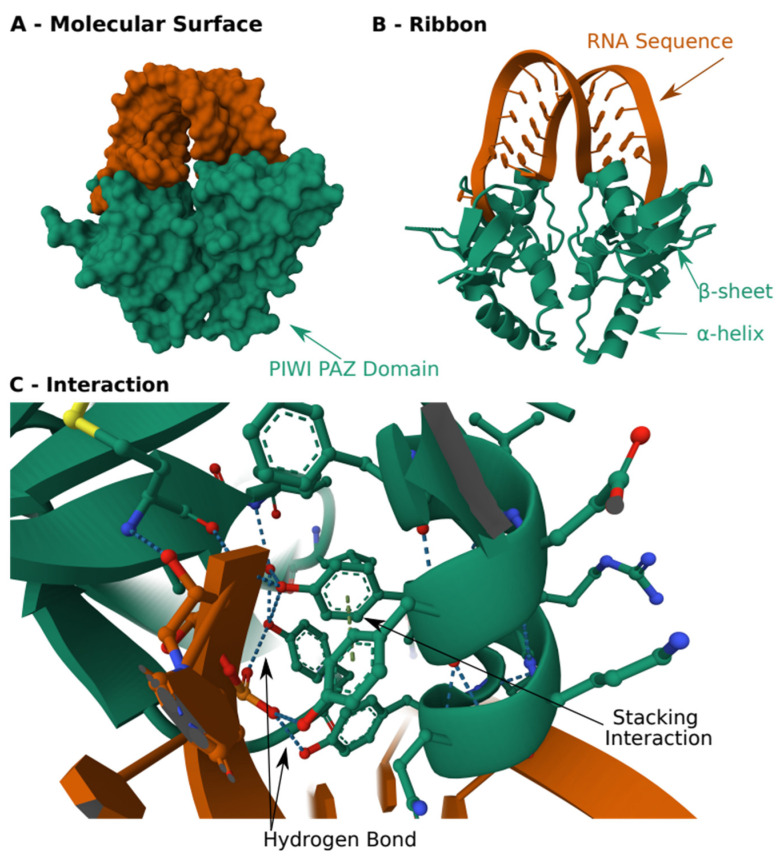
**Structure of PIWI PAZ domain in complex with RNA.** (**A**) Schematic of the molecular surface. (**B**) Schematic of arrangement of B-sheets and a-helix to recognize RNA. (**C**) Close view of the hydrogen bonds that create the binding. The structure was obtained from the protein data bank, structure: 3O3I.

Another example is the PAZ and PIWI domain adapted for RNA interference and microRNA processing that recognizes double-strand RNA similarly to DNA recognition by transcription factors [19,26] (Figure 4). These examples show how different recognition and binding methods have evolved to recognize a variety of motifs. It is also noteworthy that the rise of RNA interactome technologies made it apparent that we still underestimate the unconventional RBPs that lack known RBDs, and their investigation will most probably modify our understanding of the cellular function of RBPs [27,28].

## 3. RBPs in Vascular Disease

### 3.1. Pulmonary Arterial Hypertension

Pulmonary arterial hypertension (PAH) is a life-threatening pulmonary vascular disease that leads to increased pulmonary blood pressure, right ventricular maladaptive hypertrophy, and overtime to right heart failure and death [2,29,30]. PAH can be idiopathic, inherited, or associated with other pathological conditions [31]. Increased pulmonary blood pressure is due to the dysfunction of pulmonary vascular endothelial and smooth muscle cells. In PAH, pulmonary vascular cell dysfunction originates from a genome-wide deregulation of gene expression [32]. RBPs play a central role in gene expression regulation and thus have attracted attention in PAH [33]. Here, we describe a few examples of the critical implications of RBPs in PAH pathophysiology.

#### 3.1.1. SRSF2 Regulates BMPR2 Splicing

BMPR2 is one of the receptors of the TGFb superfamily [34]. Its mutation is the most common cause of heritable PAH, but only 20% of BMPR2 mutation carriers develop PAH. This low penetrance is due to compensatory mechanisms that prevent the phenotypic manifestation of the mutation [35]. The BMPR2 gene encodes two main isoforms, one that includes the exon 12 and the other one that excludes it. While doubt exists on the exact function of exon 12, there is no doubt that it plays a central role in BMPR2 mutation-dependent PAH [35,36,37,38,39]. Deletion of exon 12 is a common consequence of BMPR2 mutation in PAH patients, and mice over-expressing the BMPR2 isoform lacking exon 12 develop pulmonary hypertension.

The splicing of exon 12 is under the regulation of the RBP SRSF2, which promotes its inclusion [40]. In most BMPR2 mutation carriers, SRSF2 is expressed at a normal level, which promotes the inclusion of exon 12 and prevents the development of PAH. When SRSF2 expression decreases, it leads to a predominant expression of the isoform that lacks the exon 12 and to the development of PAH [40,41].

#### 3.1.2. HuR Regulates Soluble Guanylate Cyclase

Chronic hypoxia also induces pulmonary hypertension through sustained pulmonary vasoconstriction [42]. One of the early events of hypoxia exposure is the downregulation of the soluble guanylate cyclase (sGC) that produces nitric oxide—a potent vasodilator [43]. sGC stability is governed, at least in part, by the RBP HuR that stabilizes its expression. Under hypoxia, HuR is down-regulated, which results in decreased sGC expression and sustained vasoconstriction [43].

#### 3.1.3. ZFC3H1 Regulates BRD4 and HIF 1a

As part of the acquisition of genome-wide deregulation of gene expression in PAH, pulmonary arterial smooth muscle cells (PASMC) upregulate the transcription factor HIF1a [32,44] and the epigenetic reader BRD4 [45,46]. Both play a critical role in the PAH-PASMC phenotype by participating in the phenotypic switch of PASMC toward a proliferative and synthetic phenotype [31]. In PAH-PASMC, BRD4 and HIF-1a are under the RBP ZFC3H1 regulation [47]. ZFC3H1 promotes the expression of BRD4 and HIF1a in PASMC, and its inhibition leads to the down-regulation of BRD4 and HIF1a and the reversal of the PAH phenotype in a murine model of pulmonary hypertension. The mechanism by which ZFC3H1 promotes the expression of its targets in PAH is of high relevance, as ZFC3H1 is known to lead to mRNA degradation as part of the poly(A) tail exosome targeting (PAXT) connection [47,48]. Hence, these data in PASMC point towards an opposite, cell-specific function of ZFC3H1 that is yet unknown.

#### 3.1.4. HNRNPA2B1 Regulates Cell Cycle Checkpoints in PASMC

RBPs can regulate a large part of the whole cell transcriptome at once. However, up to now, in PAH, the research focused on the association between one RBP and one—or two—of its targets. A transcriptome-wide approach was missing. We started to fill this void by performing the first transcriptome-wide investigation of the role of one RBP, HNRNPA2B1, in PAH [49]. We demonstrated that in PAH-PASMC, HNRNPA2B1 regulates thousands of mRNAs at the same time. Most of these genes are implicated in cell cycle regulation—G1/S, DNA synthesis, G2/M, and Spindle assembly checkpoints, making HNRNPA2B1 a critical regulator of the PASMC phenotype. In PAH, HNRNPA2B1 was shown to be nuclear and to stabilize its targets, while in cancer HNRNPA2B1 is known to be cytoplasmic and to destabilize its targets [49]. Similar to the RBP HuR, the localization of HRNPA2B1 is crucial for its function.

### 3.2. Atherosclerosis

Atherosclerosis is the hardening and narrowing of arteries due to neointima formation, immune cell infiltration, and lipid accumulation. It is a cornerstone of cardiovascular mortality and morbidity [3]. The atherogenesis occurs in four steps: maladaptive morphological changes to endothelial cells, fatty streak formation and concomitant inflammation, proliferation of the atherosclerotic fibrous plaque and cap, and plaque destabilization [50,51]. Each step is an active cellular process regulated, at least in part, at the transcriptomic level that mediates changes in immune cells, endothelial cells, and SMC phenotypes [52]. Special interest in RBPs grows toward mitigating atherogenesis. Transcriptomic data revealed 165 differentially expressed RBPs between carotid atheroma and non-atheroma tissue, and 69 expressed RBPs between stable and unstable atherosclerotic plaques [53]. As the regulatory functions of RBPs continue to be enumerated, we will highlight a few promising applications.

#### 3.2.1. RBM47 Regulates BCLAF1

RBPs play a crucial role in the differential expression of transcription factors, specifically within their alternative splicing [51]. BCLAF1 is necessary for SMC survival and transdifferentiation to the macrophage-like/foam cell phenotype in advanced atherosclerotic plaques and is involved in the inflammatory immune response [54]. Mimicking cholesterol (ox-LDL) overload in the endothelium during atherosclerosis in vitro, an RBP RBM47-mediated, significant increase in the alternative splicing of BCLAF1 was shown, resulting in increased inflammation [51].

#### 3.2.2. Human Antigen R Regulates Endothelial Cell Preservation

Endothelial dysfunction is a precursor of atherosclerosis, as compromising the vascular barrier enables leukocyte adhesion and lipid uptake [55]. CD62E, also known as endothelial-leukocyte adhesion molecule 1 (ELAM-1), and cathepsin S, a potent cysteine protease used to degrade elastin and collagen layers on the arterial wall, are both target mRNAs of RBP Human Antigen R (HuR) on the CTSS gene [56,57]. The sulfhydration of HuR prevents its homodimerization and ability to bind to the 3′UTR of the CTSS gene [56]. As a result, CD62E and cathepsin S target mRNAs are lowly expressed and unstable, causing increased plaque formation and monocyte adherence51. Current data have shown that sulfhydration of HuR is significantly attenuated in atherosclerosis. Dietary supplementation or synthetic organic H2S donors is a promising cyto- and cardioprotective therapy [56,58,59,60].

#### 3.2.3. HuR Regulates via Competition and Cooperation

The previous examples have demonstrated the capacity of RBPs to enact specific effects via expression modulation of one or two genes. Yet, beyond its gene regulation effects mentioned earlier, the RBP HuR can interact competitively and cooperatively with several microRNAs colocalized at the same 3′UTR of the target mRNA, where competition results in enhancement or total repression—dependent on microRNA or RBP dominance—and cooperation result in low expression [61]. For example, these interactions play a role in HuR’s binding, stabilization, and cytosolic trafficking of cellular cholesterol homeostasis mRNA ABCA1, which eases foam macrophage-mediated efflux of cholesterol to circulating ApoAI and HDL [62,63]. ABCA1 mRNA is also post-transcriptionally regulated by multiple microRNAs, including MiR-33, miR-758, miR-106b, and miR-144, yet it is unclear whether HuR acts as a competitor or a cooperator in this context [61]. Still, this multi-interactional role of HuR is at the cross-section of many atherogenic pathways.

## 4. RBPs in Cardiac Disease

### 4.1. Non Ischemic Heart Failure with Reduced Ejection Fraction

Heart failure (HF) is characterized by changes in the size, shape, and function of the heart that ultimately lead to decreased cardiac output. HF is preceded by adverse cardiac remodeling which encompasses cardiomyocyte hypertrophy, cardiomyocyte death, inflammation, and fibrosis in the myocardium [64,65]. This adverse remodeling may be caused by acute stressors like myocardial infarction (MI) or chronic stressors like sustained hemodynamic overload [66]. A general upregulation of RNA-binding proteins was found in the myocardium of human and mouse HF, regardless of disease etiology [67]. During adverse cardiac remodeling and HF, genes of the fetal gene program are re-expressed. A recent study has shown that in myocardium from HF patients, expression of fetal-specific isoforms of RNA-binding proteins is reactivated together with re-expression of fetal genes. It was determined that the fetal-specific genes encode transcripts that likely contain more RNA-binding protein binding sites [68]

#### 4.1.1. CPEB4 Regulates ZEB1 and ZBTB20

A multi-omic approach in neonatal rat cardiomyocytes (NRCM) has elucidated a subset of RNA-binding proteins with altered expression and function at baseline and during pathological hypertrophic growth [69]. Of note was cytoplasmic polyadenylation element (Cpe)-binding protein 4 (Cpeb4), which was identified as an RNA-binding protein that binds and decreases the expression of Zeb1 and Zbtb20 mRNA; two essential transcription factors in cell growth. At baseline, Cpeb4-knockout mice were shown to express fetal genes, and exhibit cardiac hypertrophy and diminished cardiac function, demonstrating the powerful roles RNA-binding proteins play in disease susceptibility. Several RNA-binding proteins have been discovered to play a role in the regulation of adverse cardiac remodeling caused by non-ischemic chronic stressors.

#### 4.1.2. HNRNPC in Splicing

The heterogeneous nuclear ribonucleoprotein C (hnRNPC) upregulation was demonstrated in failing human hearts, both on whole tissue and single-cell RNA sequencing levels [67]. It was demonstrated that in diseased cardiomyocytes, mechanical stress associated with adverse cardiac remodeling causes hnRNPC to shuttle out of the nucleus. This subsequently affects the hnRNPC alternative splicing of genes involved with mechanotransduction.

#### 4.1.3. RBM38 and RBM20 Regulate Cardiac Hypertrophy

RNA-binding Motif protein-38 (RBM38) was shown to be downregulated in cardiac myoblasts in vitro upon stimulation with pro-hypertrophic stimuli such as Angiotensin II (AngII) and phenylephrine (PE). The RBM20 overexpression attenuated hypertrophy and fetal gene expression in these cells by binding and inhibiting liver X receptor (LXRα) thus reducing lipogenesis [70].

#### 4.1.4. CELF1 Activates MAPK

CUG triplet repeat-binding protein 1 (CELF1) was upregulated in mouse hearts upon pressure overload induced by transverse aortic constriction (TAC), while CELF1-KO mice exhibited attenuated cardiac hypertrophy and fibrosis, and improved cardiac function upon pressure overload. In NRCM stimulated with AngII, CELF1 was upregulated and shown to directly bind RNA encoding for phosphatidylethanolamine-binding protein 1 (PEBP1), thereby activating Raf/MAPK signaling [71]. As such, CELF-deficient cardiomyocytes were more resistant to AngII-induced apoptosis, which was recapitulated in CELF-KO mouse hearts.

#### 4.1.5. REGnase Family in Inflammation

The Regnase family RNA-binding proteins play important immunomodulatory roles by regulating the degradation of proinflammatory cytokine mRNAs [72]. Cardiomyocyte-specific Regnase-1–knockout mice showed severe cardiac fibrosis and immune cell infiltration, as well as dilated cardiomyopathy and HF upon TAC-induced pressure overload [73]. It was revealed that a higher number of cardiomyocytes express interleukin 6 (Il6) mRNA, but not other cytokines, in cardiomyocyte-specific Regnase-1–knockout mouse hearts upon pressure overload. Accordingly, the administration of an anti–Il6 receptor antibody attenuated cardiac inflammation and cardiomyopathy in cardiomyocyte-specific Regnase-1–knockout mice.

### 4.2. Myocardial Infarction and Cardiac Ischemia-Reperfusion Injury

MI due to atherosclerotic or thrombotic occlusion of coronary arteries leads to ischemia in the myocardium. While rapid reperfusion is the cornerstone of current MI treatment, restoring blood flow leads to further damage to the myocardium by causing ischemia-reperfusion injury (IRI) [4]. An IRI is characterized by oxidative stress, cell death, inflammation, and fibrosis. A delicate interplay between these processes underlies the balance of tissue damage and tissue repair and thus disease outcome. RNA-binding proteins may govern this interplay.

A study leveraging four large datasets of mouse and human MI and HF found that up to 17% of genes that are upregulated in diseased compared to control myocardium are annotated as having RNA-binding ontology [67]. In human hearts from ischemic HF patients, the RNA-binding proteins DDX52, RRS1, FCF1, DHX15, POLR1D, GNL2, RSL24D1, and EBNA1BP2 were found to be downregulated as compared to control hearts, while LSG1 and GRWD1 were upregulated in ischemic HF myocardium. Additionally, a recent study in mouse IRI myocardium demonstrated differential expression of RNA-binding proteins together with important alterations in splicing events of genes in pathways related to myocardial function [74].

#### 4.2.1. HuR Regulates Beclin1

The functional roles of a few specific RNA-binding proteins in MI or IRI have been reported thus far. The RNA-binding protein ELAV-like protein 1 (ELAVL1) also known as human antigen R (HuR), is upregulated in mouse myocardium upon IRI as well as in cardiomyocytes subjected to hypoxia-reoxygenation in vitro [75]. ELAVL1 promoted reactive oxygen species (ROS) and reduced myocyte viability by directly binding and stabilizing Beclin1 mRNA, thus promoting ferroptosis, an iron-dependent form of cell death.

#### 4.2.2. METTL3 Regulates PTBP1

The RNA-binding protein methyltransferase-like 3 (METTL3), a subunit of the methyltransferase complex, was upregulated in the myocardium of mice with chronic, non-reperfused, MI [76]. Cardiac-specific METTL3 silencing attenuated cardiac fibrosis and improved cardiac function and contractility upon MI. Another upregulated RNA-binding protein in mouse MI myocardium is polypyrimidine tract binding protein 1 (PTBP1) and cardiac LV-specific silencing of PTBP1 resulted in reduced MI-induced fibrosis in mice [77].

### 4.3. Cardiac Fibroblasts and Fibrosis

Cardiac fibrosis is characterized by excessive extracellular matrix production and is observed in numerous cardiac disorders where cardiac fibrosis critically contributes to disease progression by impairing myocardial compliance, contractility, and electrical conductance [5]. A key pathophysiological event in cardiac fibrosis is the activation of cardiac fibroblasts (CF) and subsequent differentiation into myofibroblasts. Recently, it was demonstrated in CF explanted from human cardiac biopsies that about one-third of all gene expression changes in activated CF are subject to posttranslational regulation and that these genes are strongly enriched for targets of RNA-binding proteins [78].

#### 4.3.1. PUM2, QKI and MBNL1 Regulate Myofibroblast Differentiation

Data on RNA-binding proteins in myofibroblast differentiation have started emerging. In vitro cell studies have demonstrated several RNA-binding proteins to play roles in myofibroblast differentiation. Silencing of Pumilio RNA-binding family member 2 (PUM2) and Quaking (QKI) in explanted human CF inhibited TGFβ-induced myofibroblast differentiation as characterized by lower expression of ACTA2 and MMP2 secretion [78].

RNA-binding protein muscleblind-like1 (MBNL1) was also shown to promote myofibroblast differentiation in neonatal mouse CF by directly binding a network of differentiation and matrix-assembly transcripts, including the transcriptional regulators serum response factor (SRF) and twist, as well as TGFβ receptors I and II76. Accordingly, MBNL1 overexpression promoted ANGII/PE-induced cardiac fibrosis in mice [79].

#### 4.3.2. TIAR and LncRNAs

The RNA-binding protein TIA1-related protein (TIAR), under the control of the long non-coding RNA Wisper, was shown to control the expression of a profibrotic form of lysyl hydroxylase 2 (PLOD2) in adult mouse CF77. Previously, PLOD2 was implicated in collagen cross-linking and ECM stabilization. The RNA-binding protein HuR was shown to bind a duplex consisting of long non-coding RNA Safe and SFRP2 RNA, enhancing their stability [80]. HuR silencing in adult mouse CF downregulated Safe-SFRP2 expression and thereby inhibited TGF-β-induced myofibroblast differentiation. RNA-binding protein PTBP1 promotes TGF-β1-induced collagen accumulation and proliferation in Cfs [77]. PTBP1 was shown to bind nuclear receptor Nur77 mRNA to destabilize its expression, subsequently inhibiting the fatty acid-binding protein 5 (FABP5) pathway and thereby myofibroblast differentiation.

Demonstrating the powerful roles RNA-binding proteins play in disease susceptibility, in vivo, it was shown that RNA-binding motif 24 (RBM24) overexpression in otherwise-healthy mice was sufficient to cause extensive cardiac fibrosis, as well as expression of genes associated with the immune response and myofibroblasts activation, demonstrating the powerful roles RNA-binding proteins play in disease susceptibility [81].

Non-classical RNA-binding proteins have also been implicated in cardiac fibrosis. The non-classical RNA-binding protein cytoskeleton-associated protein 4 (CKAP4) was downregulated in the fibrotic right atrium in patients with cardiac valvular disease compared to healthy atrial tissue. Bioinformatics revealed that CKAP4 preferentially binds to long non-coding RNAs (lncRNAs) in the fibrotic atrium than in normal atrial tissue. CKAP4 was experimentally validated to have stronger binding to lncRNAs LINC00504, FLJ22447, RP11-326N17.2, and HELLPAR in fibrotic atrium compared to control atrial tissue80. The non-classical RNA-binding protein METTL3 was demonstrated to promote TGFβ-induced CF proliferation and myofibroblast transition by modifying N6-methyladenosine on fibrosis-related RNA transcripts, such as fibroblast growth factor 2 (FGF2), fibronectin (FN1), and several collagen isoforms [76].

### 4.4. Dilated Cardiomyopathies

Cardiomyopathies are myocardial disorders characterized by structural and functional abnormalities [6]. In dilated cardiomyopathy (DCM), the cardiac left ventricle is enlarged and exhibits poor contractility, causing DCM to be a major HF risk factor. Both genetic and non-genetic causes may underlie DCM. It was recently shown that natural genetic variations influence translational regulation in human DCM myocardial tissue and that these genetic variations were predicted to affect RNA-binding protein function [82]. One of the best-characterized RNA-binding proteins in DCM is RNA-binding motif protein 20 (RBM20), with ample clinical and experimental data to support its crucial role. Mutations in RBM20 are known to cause highly penetrant DCMs. RBM20 mutations are thought to explain up to 2–6% of all genetic DCM cases [83]. RBM20 is a splicing regulator with over 30 validated splicing targets to date [84]. In particular, RBM20 regulates splicing of sarcomeric genes (TTN, FHOD3, MYOM1), but also calcium handling and signaling (CACNA1C, RYR2, CAMK2D) and contraction (TNNT2). Miss splicing of Titin (TTN), a Z disc protein crucial for myocardial contraction, is thought to be one of the primary underlying mechanisms of RBM20-associated DCM. Similarly, RNA-binding motif protein 24 (RBM24) regulates a subset of genes associated with cardiomyopathies such as sarcomere z-disc genes TTN, nebulette (NEBL), and enah actin regulator (ENAH).

Global splicing profiling showed that RBM24 regulated more than 500 isoform switches in mouse DCM hearts [85]. Accordingly, cardiomyocyte-specific RBM20-knockout mice exhibited rapidly progressive DCM and HF85. Another study performing translatome profiling in the hearts of DCM patients revealed that key network hubs include the RNA-binding proteins Pumilo family member 2 (PUM2) and Quaking (QKI), however, their functional role was not assessed [78].

### 4.5. Right Ventricular Disease

**Figure 5:**
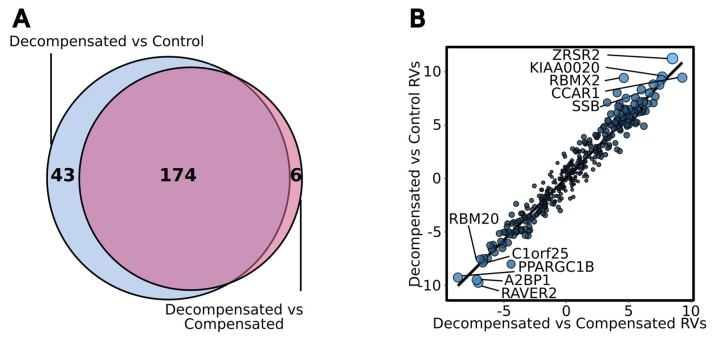
**RBP expression in Compensated and Decompensated human right ventricle.** (**A**) Venn diagram showing differentially expressed genes (DEGs) coding for RNA-binding proteins overlapping between two human RV comparisons: decompensated PAH vs. control, and decompensated PAH vs. compensated PAH. DEGs were determined by DESeq2. (**B**) Scatter plot showing correlation of Wald statistic of genes coding for RNA-binding proteins from DESeq2 DEG analysis between two human RV comparisons: decompensated PAH vs. control (*y*-axis), and decompensated PAH vs. compensated PAH (*x*-axis). Higher absolute Wald statistic indicates stronger differential expression. Larger size and lighter blue dots indicate stronger false discovery rate-corrected *p*-values (combined by Wilkinson’s method between the two comparison). Select genes are labeled.

While the role of RNA-binding proteins has begun to surface in left ventricular disease, little is known about their role in right ventricular (RV) disease. Failure of the right ventricle is an important predictor of morbidity and mortality in a number of diseases affecting the heart and/or lungs such as PAH [86], yet there have been no studies investigating RNA-binding proteins in right ventricular disease, to our knowledge. The increasing availability of genome-wide datasets allows for unbiased interrogation of RNA-binding proteins, especially for tissues that may be difficult to procure for research. In the largest study to date in human right ventricular tissue from patients with PAH [87], Boucherat et al. performed multiomic profiling of RV tissues including transcriptomics of seven decompensated and 11 compensated RVs from PAH patients and 14 control RVs. They identified a panel of proteins upregulated in both RV and plasma of patients with PAH with decompensated RVs and further identified latent transforming growth factor beta binding protein 2 (LTBP-2) as a novel plasma biomarker in PAH that correlates with RV function and predicts long-term survival. We interrogated their transcriptome-wide dataset to identify RNA-binding proteins that might be dysregulated in PAH RVs. We intersected 415 RBPs from the RBP database with differentially expressed genes (DEGs) from this dataset and found that 217 RBPs were dysregulated (FDR < 0.05) in decompensated PAH RVs vs. control RVs and 180 RBPs were dysregulated (FDR < 0.05) in decompensated PAH RVs vs. compensated PAH RVs, with 174 RBP DEGs overlapping and directionally consistent between these two comparisons. These findings suggest that RBPs may play an important role in the RV and that their dysregulation is associated with RV disease, such as decompensated RV function in patients with PAH. While most of the top RBP DEGs have not been previously implicated in RV disease, RBM20 was a top downregulated DEG that has been reported to have a link to RV disease, in addition to its well-described role in DCM of the LV discussed above (Figure 5). Specifically, Malakootian et al. [88] performed whole-exome sequencing to identify a pathogenic variant in RBM20 in a family with heritable DCM. Cardiac magnetic resonance imaging of one of the affected family members revealed an enlarged right ventricle with reduced right ventricular function. This report suggests that mutations in RBM20 may be pathogenic for not only LV disease but also RV disease. However, further studies are needed to investigate the role of RBM20 and other RBPs in RV disease.

## 5. Therapeutic Strategies

Because RBPs regulate every aspect of transcripts’ life, they are attractive therapeutic targets. We use three main strategies to target them: Small interfering RNA, small molecules, and Nucleotide aptamer. All these approaches are at the pre-clinical stage in cardiovascular disease despite being at the clinical stage in numerous other diseases.

Si-RNA attracted a lot of attention as they have the innate advantage of being quick to develop compared to small molecules. Nonetheless, in their native form, they have poor stability and pharmaco-kinetic [89]. Numerous modifications of Si-RNA are now known to improve stability and pharmacokinetics and open their use as a therapy. A few Si-RNA are in clinical trials for cardiovascular diseases, though none targets RBPs yet [90].

Small molecules are the main therapeutic tools of modern medicine. Numerous have already been developed to target RBPs and are currently in clinical trials, mainly in cancer. For example, small molecules bind to the RBP HuR (MS-44, Okicenone, and Dehydromutacin) [91] are in pre-clinical trials in liver diseases [92]. In cancer, Hippuristanol—an inhibitor of the EiF4A is a promising anti-neoplasmic agent [93]. In addition, splieceostatin A and other SF3b inhibitors have attracted attention as anti-cancer drugs. SF3b is a subunit of the spliceosomal U2 small nuclear ribonucleoprotein. Its inhibition prevents proper splicing and leads to massive nonsense-mediated decay [94] and cell death.

Another approach uses an aptamer that mimics the motif recognized by the RBP [95]. The RBP will bind to the aptamer and become inactive. This approach has the advantage of only targeting the function of the RBP linked to its binding to one specific motif. RBP can recognize multiple motifs and/or different modifications, and Si-RNA or small molecules could alter other unintended functions of the RBPs. Again, no clinical trials using the aptamer are underway in cardiovascular diseases, but numerous clinical trials are ongoing in other diseases [96].

The examples above show that RBPs are relevant therapeutic targets in many diseases, but their use in cardiovascular diseases is still at its dawn. Before we can harvest their potential, we need a better understanding of their specific function.

## 6. Perspectives

Our understanding of the role of RBPs in cardiovascular health and disease development is at its infancy. A few limitations may block the natural course of RBP therapies.

One is the context-dependent function of RBPs. Among all the potential targets of an RBP, the ones expressed by a cell at a given point will determine the role of the RBP on the cell phenotype. One other layer of complexity is that an RBP has no activity per se, but is part of an enzymatic complex (recruitment of the ribosome, the CCr4-Not complex, etc.). One RBP can be part of multiple complexes and thus lead to different end-points for a given pool of mRNAs. For example, HNRNPA2B1 in healthy pulmonary arterial smooth muscle cells is cytoplasmic and leads to mRNA degradation. However, in PAH pulmonary arterial smooth muscle cells, HNRNPA2B1 is nuclear and promotes the stability and expression of its targets [49].

The approaches we used so far also greatly limited our understanding. Much of the work focused on a limited number of targets because of limited access to transcriptomic data, bioinformatic tools, and the computational power needed for transcriptome-wide analysis of their function. However, to harvest the full therapeutic potential of RBPs, it is necessary to go beyond individual targets [49]. The advent of single-cell RNA-seq is a promising avenue to decipher the cell and disease specificity of RBPs function and thus refine our use of RBPs for therapeutic purposes. One limitation, though, is that single-cell RNA-seq depth is not as deep as bulk RNA-seq and RBPs as transcription factors mRNAs tend to be kept at a low transcript number to maintain a tight control over their protein expression. Nonetheless, technologies are evolving faster than ever and this limitation may soon be removed. 

We can expect that the therapeutical use of RBP will, in the long term, greatly improve life quality and survival, as they can specifically target a given pool of mRNA and influence the entire transcriptome.

## 7. Conclusions

Here, we summarize some of the noteworthy evidence presented for the role of RBPs in systemic and pulmonary cardiovascular diseases. We also highlight the complexity of investigating RBPs mechanism of action, as they have a highly cell-specific and context-specific function. We are just beginning to understand the function of RBPs in the cardiovascular system and much remains to be discovered before we grasp the full scope of RBP regulation.

## Figures and Tables

**Table 1:** Summary of RBPs, their targets, and the resulting phenotype.

Disease	RNA-Binding Protein	Targets	Phenotype
Pulmonary Arterial Hypertension	SRFS2	BMPR2 Splicing	Pathogenesis
	HuR	Guanylate Cyclase	Vasoconstriction
	ZFC3H1	BRD4 and HIF1a	Proliferation
	HNRNPA2B1	Cell Cycle Checkpoints	Proliferation
Atherosclerosis	RBM47	BCLAF1	Inflammation
	HuR	Cathepsin S	Plaque Formation
	HuR	Competition and Cooperation with miRs	Foam Macrophages
Non Ischemic Heart Disease	CPEB4	ZEB1 and ZEBTB20	Cell Growth
	HNRNPC	Splicing	Failing Heart transcriptome modification
	RBM20		Hypertrophy
	CELF1	MAPK	Resistance to Apoptosis
	REGnase	Cytokines	Inflammation
Myocardial Ischemia & Ischemia Reperfusion Injury	HuR	Belcin1	Apoptosis
	METTL3	PTBP1	Fibrosis
Cardiac Fibrosis	PM2/QKI	ACTA2 and MMP2	Myofibroblast differentiation
	MBNL1	SRF	Myofibroblast differentiation
	TIAR	LncRNA Wisper	Fibrosis
Dilated Cardiomyopathy	RBM20 mutation	Splicing	High penetrant DCM
	RBM24	ENAH	High penetrant DCM
Right Ventricular Hypertrophy	RBM20	Unknown	Unknown
